# The Intraosseous Environment: Physiological Parameters, Regulatory Mechanisms, and Emerging Insights in Bone Biology

**DOI:** 10.3390/ijms26083876

**Published:** 2025-04-19

**Authors:** Mikhail Yu. Artamonov, Evgeniy L. Sokov, Lyudmila E. Kornilova, Inessa A. Minenko

**Affiliations:** 1 Department of Physical Medicine and Rehabilitation, Penn Medicine Princeton Health, Plainsboro, NJ 08536, USA; 2 Department of Algology, Peoples’ Friendship University, Moscow 117198, Russia; 3 Department of Sports Medicine, Sechenov Medical University, Moscow 119991, Russia; minenko_i_a@staff.sechenov.ru

**Keywords:** intraosseous therapy, hematopoiesis, regeneration, bone marrow environment, mesenchymal stem cells, mechanotransduction

## Abstract

The intraosseous environment is a dynamic and intricate system integral to bone health, encompassing vascular, cellular, and biochemical interactions that drive key processes such as hematopoiesis, bone remodeling, and systemic mineral regulation. This review examines the structural composition of the bone matrix, the diverse cellular landscape, and the interconnected vascular and nervous networks, highlighting their roles in preserving bone function and responding to pathological changes. Recent studies reveal regulatory mechanisms involving oxygen tension, ionic balance, signaling molecules, and mechanotransduction pathways that shape bone metabolism and its adaptation to mechanical forces. Insights into the bone microenvironment’s metabolic shifts in cancer and its interaction with inflammation underscore its pivotal role in disease progression and therapeutic innovation. Additionally, advances in imaging techniques and biomaterials fuel progress in bone regeneration and understanding its microenvironment. Exploring the intricate physiochemical dynamics and regulatory networks within the intraosseous system unlocks potential clinical applications in bone diseases, tissue engineering, and systemic metabolic disorders. This comprehensive review bridges fundamental science with translational research, offering insights into the complex yet essential intraosseous environment.

## 1. Introduction

The intraosseous environment refers to the internal space within bones, encompassing the bone marrow, blood vessels, and the specialized microenvironment that supports bone cells like osteoblasts, osteoclasts, and osteocytes. In medical emergencies, intraosseous access is often used to deliver fluids and medications directly into the bone marrow, offering a rapid pathway to the bloodstream. This technique can save valuable time for critically ill patients, significantly reducing delays in medication delivery. In prehospital settings, traditional manual intraosseous infusion devices have shown higher success rates and faster insertion times compared to semi-automatic devices.

Beyond its medical applications, the intraosseous environment plays a crucial role in maintaining skeletal health and function. Rather than acting as a passive framework, it supports dynamic biological processes such as bone remodeling and hematopoiesis. The bone tissue’s dense vascularization facilitates the exchange of nutrients, hormones, and metabolic waste. Recent studies suggest a close association between vascular health and bone density, indicating that impaired blood flow may contribute to weakened bone structure and increased fracture risk. This complex vascular skeleton interface is increasingly recognized as essential for preserving bone integrity and supporting broader physiological functions [1]. Additionally, the presence of pain associated with metastatic disease in the bone highlights the critical role of the intraosseous environment in cancer-related conditions. Investigating the peripheral mechanisms influencing intraosseous pathways, particularly the role of bone marrow afferent neurons, offers valuable insights into pain management. Understanding these pathways could lead to innovative therapeutic approaches, aiming to alleviate pain and improve the quality of life for patients dealing with bone metastases [2]. The intraosseous environment must be recognized as a dynamic system essential to both skeletal physiology and clinical outcomes. Research indicates that the bone microenvironment is integral to bone regeneration, maintaining homeostasis, immune responses, and cancer progression. Bone serves as a supportive microenvironment for diverse cell types that collectively orchestrate vital skeletal functions, including energy metabolism, mineral regulation, bone formation, and blood cell production.

Endothelial cells form a complex vascular network within the bone which supports and organizes a distinct microenvironment. Recent findings on vascular heterogeneity in the bone marrow reveal multiple specialized vascular niches, each characterized by unique cellular and molecular compositions. These niches offer specific regulatory cues essential for physiological processes such as hematopoiesis and bone remodeling. However, emerging studies suggest that systemic and environmental factors including those originating from the endobronchial environment such as inflammatory mediators or hypoxic conditions may influence vascularization through their impact on endothelial cell function. These interactions highlight the dynamic crosstalk between distant organ systems and the bone vasculature with implications for both health and disease. This intricate interplay underscores the importance of the intraosseous environment in maintaining bone health and responding to systemic and local challenges [3]. Glycosaminoglycans play a pivotal role in shaping the bone microenvironment by interacting with key mediators of various signaling pathways. These interactions can significantly influence the activity and function of bone-remodeling cells, such as osteoblasts, osteoclasts, and osteocytes. By modulating these pathways, glycosaminoglycans contribute to the regulation of bone remodeling, impacting processes like bone formation, resorption, and overall skeletal homeostasis [4]. Bone provides a unique metabolic microenvironment, home to highly energy-intensive processes such as bone resorption and bone formation, which are dysregulated in the presence of cancer. Approaches such as metabolomics demonstrate metabolic plasticity in patients with advanced diseases. Metabolic crosstalk between tumor cells and the surrounding stroma supports disease pathogenesis. Increasing evidence suggests that metabolic reprogramming within the tumor–bone microenvironment plays a key role in driving disease progression. Furthermore, understanding these metabolic adaptations will reveal new therapeutic targets and approaches [2].

This review explores the core factors influencing the intraosseous environment including oxygen levels, nutrient availability, and signaling molecules. By examining these elements, we can gain deeper insight into their contribution to bone health and systemic functions, paving the way for a more comprehensive understanding of the intraosseous microenvironment. In addition to these factors, current studies have highlighted the significant role of cell scaffold materials in biochemical activation mechanisms that support tissue regeneration and cellular behavior. For example, bioactive scaffolds incorporating potassium ions have been shown to influence osteogenic differentiation, modulate inflammation, and enhance vascularization. Materials such as potassium-doped bioglass and potassium-containing hydrogels are gaining attention for their ability to release ionic cues that activate specific cell-signaling pathways critical for bone repair and regeneration. Including such examples enriches our understanding of how engineered materials interact with the bone microenvironment at both the cellular and molecular levels [5].

## 2. Structural Composition of the Intraosseous Environment

The intraosseous environment is a critical hub for processes like blood cell production (hematopoiesis), bone remodeling, and pain regulation. A major factor shaping this environment is the composition of bone marrow, which includes both hematopoietic and mesenchymal stem cells. Recent research has revealed changes in gene expression related to pain pathways in the peripheral nervous system, highlighting the role these cells play in managing pain within the bone. Molecular signaling further adds to this complexity, with osteoclasts being closely linked to bone pain. Interestingly, the balance of osteoclast activity appears finely tuned only when their activity surpasses a certain threshold, triggering inflammation and pain. This delicate regulation underscores the intricate dynamics of the intraosseous environment. Gaining a deeper understanding of these factors is crucial for designing targeted therapies for conditions like osteoarthritis and cancer-related bone pain, ultimately improving patient outcomes [6].

The bone’s structural composition changes substantially during various life stages and fracture healing, and in diseases such as osteoporosis. In childhood, bone is very dynamic with high growth and mineralization, causing an increase in bone mass and density. As people grow older, especially post-middle age, bone remodeling decreases and bone resorption exceeds bone formation, causing a reduction in bone mass and structural compromise. In osteoporosis, this imbalance is increased, resulting in porous and weak bones, particularly in the spine, hips, and wrists. In fracture healing, the bone matrix reorganizes temporarily with a soft callus formation, followed by a hard callus, as the fracture stabilizes, eventually remodeling into mature bone. These structural changes mirror the bone’s reaction to mechanical stress, aging, injury, and metabolic disease, affecting its strength, function, and capacity for healing.

### 2.1. Bone Matrix Components

**Figure 1:**
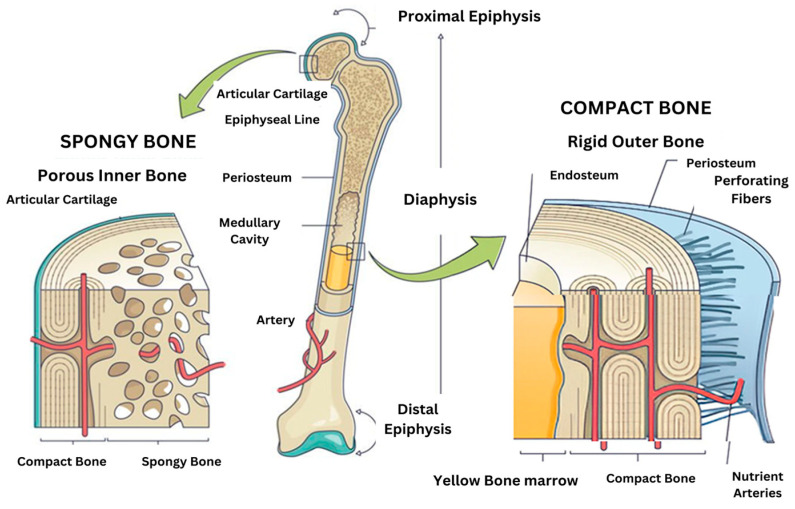
Structure of a long bone showing spongy and compact bone with their anatomical components.

The bone matrix is made up of several key components, including organic proteins (primarily type I collagen), and inorganic minerals like hydroxyapatite, and a variety of extracellular matrix proteins that help regulate bone remodeling and regeneration. Bone mineralization is a complex process that begins with calcium-phosphate-loaded vesicles inside cells, which are thought to serve as precursors for the formation of carbonated hydroxyapatite. This intricate system ensures the strength, structure, and adaptability of bones [1]. Bone minerals are connected to the organic matrix through protein-bound phosphate bonds [7]. These bonds are an integral part of both the structural organic matrix and the inorganic calcium phosphate crystals, creating a strong and cohesive framework that supports bone strength and function. The cellular composition of two microenvironments (spongy bone and compact bone) are different in Figure 1. Despite extensive research, some aspects of the composition and structure of mature bone mineral particles remain unclear. These particles are often described as calcium-deficient and hydroxyl-deficient carbonated hydroxyapatite, with some of the PO_4_^3−^ lattice sites occupied by HPO_4_^2−^ ions. Using advanced solid-state nuclear magnetic resonance techniques, researchers have closely examined the hydrogen-bearing components in bone minerals, particularly focusing on the presence of HPO_4_^2−^ ions, to gain deeper insights into their role in bone structure [3].

### 2.2. Organic Phase (Collagen and Non-Collagenous Proteins)

Bone consists of a specialized calcified extracellular matrix, which is a primary source of connective tissue components in the body. The organic matrix is predominantly composed of type I collagen (90%), which is distinct from type I collagen in other connective tissues due to unique post-translational modifications. The remaining 10% of the organic matrix is composed of non-collagenous proteins, which have been extensively studied over the past 15 years, aided by advancements in protein biochemistry and molecular biology, as reviewed by Gehron-Robey (1989) [8].

Through the use of dissociating agents (e.g., EDTA) and non-dissociating buffers, researchers have been able to differentiate the mineral phase (hydroxyapatite crystals) from the organic phase (collagenous matrix) and analyze the distribution of proteins between these phases. The proteins in the bone matrix are classified as either exogenous or endogenous. Exogenous proteins are synthesized in other organs, circulate in the blood and tissue fluids, and become incorporated into the bone matrix due to their affinity for hydroxyapatite. Endogenous proteins, produced by osteoblasts, are directly integrated into the three-dimensional structure of the bone matrix during its formation. These bone matrix proteins are of significant interest due to their critical roles in regulating various aspects of bone physiology and remodeling [4].

### 2.3. Cellular Landscape

The cellular composition of bones includes several key cell types, each contributing uniquely to bone formation, remodeling, and maintenance. These include osteoblasts, osteoclasts, osteocytes, and bone marrow stromal cells. Osteoblasts are specialized cells responsible for synthesizing the mineralized matrix of bones. They arise from multiple sources, such as chondrocytes within the growth plate, bone marrow stromal cells, quiescent bone lining cells, and specific fibroblasts in craniofacial regions. With a finite lifespan, osteoblasts require continuous renewal by preosteoblasts, their direct precursors. The differentiation of osteoblasts from skeletal stem cells is tightly regulated by signaling pathways, including the non-canonical Notch molecule Delta-like 1/preadipocyte factor 1 (Dlk1/Pref-1) and the Wnt co-receptor Lrp5 [9].

**Figure 2:**
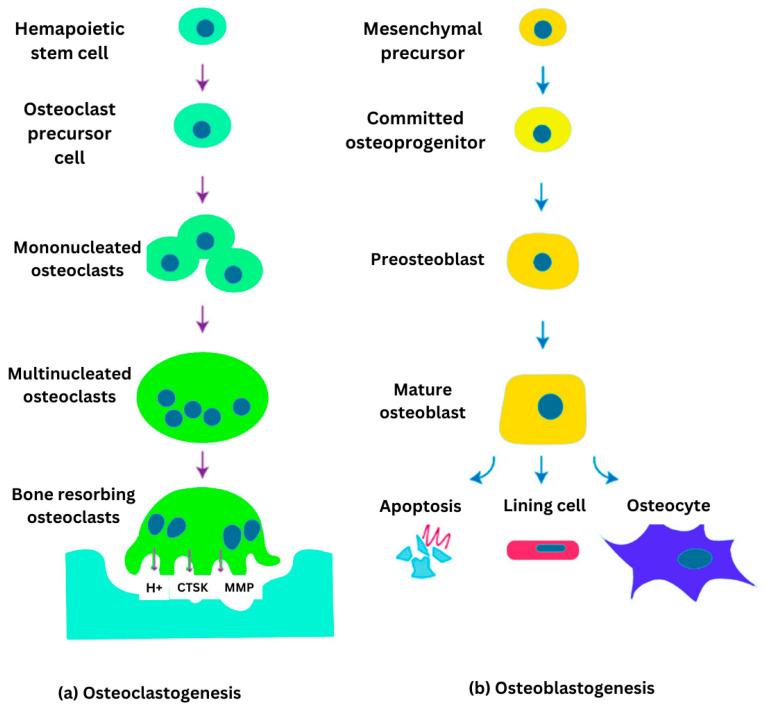
Illustration of bone cell differentiation: (**a**) osteoclastogenesis from hematopoietic stem cells leading to bone resorbing osteoclasts; and (**b**) osteoblastogenesis from mesenchymal precursors forming mature osteoblasts which further differentiation into lining cells or osteocytes.

Bone cell differentiation osteoclastogenesis and osteoblastogenesis are illustrated in Figure 2. Osteoclasts are large, multinucleated cells specialized in bone resorption. They degrade bone tissue by secreting acids and enzymes that dissolve the mineralized matrix and collagen. This process plays a vital role in bone remodeling and the regulation of calcium levels in the body. Osteoclast activity is meticulously controlled by osteocytes and osteoblasts through diverse signaling molecules, ensuring the maintenance of bone homeostasis [10]. Osteocytes are osteoblasts that become embedded within the mineralized bone matrix. These dynamic and multifunctional cells act as central regulators, integrating hormonal and mechanical signals to coordinate the activity of osteoclasts and osteoblasts. Osteocytes are essential for maintaining bone homeostasis and influence factors such as bone marrow fat, body composition, and energy metabolism through both paracrine and endocrine signaling. Additionally, they play a key role in the development of various bone diseases and serve as promising targets for emerging therapeutic strategies [11].

Bone marrow stromal cells, also referred to as skeletal stem cells, are multipotent cells located within the bone marrow stroma. These cells can differentiate into osteoblasts, chondrocytes, and adipocytes. BMSCs are critical for bone formation and regeneration, with their differentiation into osteoblasts being regulated by various intracellular signaling pathways. Additionally, under specific genetic conditions, these cells are linked to the development of osteosarcoma, a primary malignant bone tumor [12]. All of them are summarized in Table 1.

### 2.4. Vascular and Nervous Network Within Bone

The vascular and nervous networks within bone are essential for its development, maintenance, and repair. These systems deliver critical nutrients, oxygen, and regulatory signals that support bone health and regeneration. The bone vasculature comprises a dense network of blood vessels, including arteries, arterioles, and capillaries, which provide oxygen, nutrients, and growth factors to bone cells. This intricate network plays a vital role in bone development, remodeling, and regeneration [13]. The endothelial niche plays a crucial role in maintaining hematopoietic stem cells (HSCs) by regulating their quiescence, proliferation, and differentiation through complex molecular and cellular interactions. Recent studies emphasize the role of specialized vascular structures, including sinusoidal and arteriolar networks, in creating distinct microenvironments for HSC regulation. Arteriolar vessels are associated with quiescent HSCs, while sinusoidal vessels facilitate HSC mobilization and differentiation. Furthermore, the interaction between endothelial cells, perivascular stromal cells, and extracellular matrix components provide key regulatory signals through pathways such as VEGF, Notch, and CXCL12. These molecular cues coordinate HSC maintenance, mobilization, and immune function. Disruptions in endothelial niche function have been implicated in hematological disorders, impaired immune responses, and defective bone regeneration, underscoring its physiological significance [14,15]. The pathological implications of vascular network dysfunction include the development of conditions such as ischemia, arthritis, osteonecrosis, and osteoporosis. Adequate vascularization is essential for successful outcomes in bone grafting and tissue engineering, underscoring the importance of a healthy vascular system in maintaining bone integrity and supporting repair processes [16].

Nerve fibers are widely distributed throughout the bone, with a particular concentration in the periosteum, cortical bone, and cancellous bone. These fibers play a vital role in bone formation, remodeling, and repair by closely interacting with the vascular network, highlighting their importance in maintaining bone health and facilitating regeneration [13]. The interaction between nerve fibers and blood vessels, termed neurovascular coupling, enhances the development and function of both systems. This coupling is essential for effective bone regeneration and the integration of bone implants [17]. Nerve fibers are most densely concentrated above bone remodeling surfaces and within cortical pores, emphasizing their critical role in the bone remodeling process. Their close association with vascular structures, particularly capillaries and arterioles, highlights the intricate interplay between the nervous and vascular systems in regulating bone health and remodeling [18].

**Figure 3:**
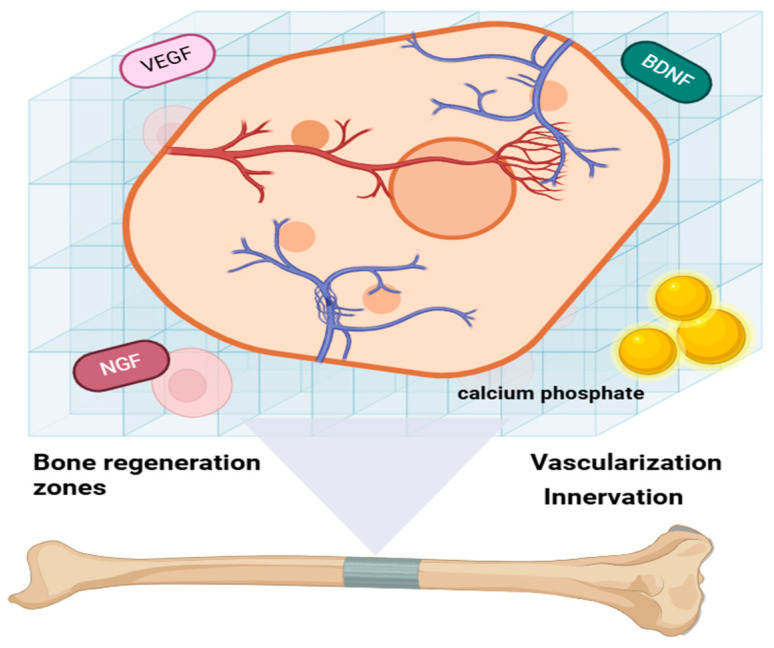
3D schematic representation of advanced bone biomaterials: polyhedron-like scaffolds integrated with vascular and nervous networks supported by hydrogel matrices, illustrating their role in promoting osteogenesis, angiogenesis, and neurogenesis for enhanced bone regeneration.

Integrating vascular and nervous networks into bone biomaterials presents significant challenges but is essential for replicating the natural bone environment and enhancing bone regeneration. Recent advancements in this field include the development of innovative hydrogels and polyhedron-like scaffolds designed to promote both angiogenesis and neurogenesis, offering promising solutions for improving bone repair and regeneration [19], as indicated in Figure 3. Hydrogels exhibit excellent biocompatibility and can be tailored to support both vascularization and innervation. This adaptability makes them highly promising candidates for applications in bone regeneration, as they effectively mimic the native bone environment and facilitate repair processes [20]. Polyhedron-like scaffolds, which are 3D-printed to replicate the structure of cancellous bone, have demonstrated the ability to enhance osteogenesis, angiogenesis, and neurogenesis. By mimicking the native bone architecture, these scaffolds significantly improve bone regeneration outcomes, making them a valuable innovation in tissue engineering. Bone biomaterials may be generally divided into natural and synthetic materials, including calcium phosphates, bioactive glasses, and hydroxyapatite-based composites. The hydroxyapatite (calcium phosphate) that closely resembles the mineral phase of the natural bone is of special importance because of its high biocompatibility, osteoconductivity, and chemical resemblance to native bone minerals. Their chemical structure has a profound impact on their biological behavior; for example, doping HA with trace ions such as magnesium, strontium, or zinc can improve its bioactivity and promote remineralization. These materials have a pivotal function in orthopedic applications through supporting bone ingrowth, matrix deposition, and long-term host tissue integration, rendering them a cornerstone in bone tissue engineering and regenerative medicine.

### 2.5. Bone Biomaterial

Bone biomaterials play a pivotal role in tissue engineering strategies aimed at promoting bone regeneration [21]. While the foundational principle involves combining cell scaffold materials with growth factors to guide cellular activity, recent advancements have led to the development of a diverse array of biomaterials with tailored properties to mimic the native bone environment more closely [22]. Bone biomaterials can be broadly categorized into natural, synthetic, and composite materials. Natural materials such as collagen, gelatin, and chitosan offer excellent biocompatibility and bioactivity, but they often lack mechanical strength. Synthetic materials such as polylactic acid, polyglycolic acid, and polycaprolactone offer tunable mechanical properties and degradation rates, though they may require surface modification to enhance cell adhesion [23]. Among inorganic materials, calcium-phosphate-based ceramics have strong osteoconductivity and are widely used in both clinical and research settings. Recent innovations include ion-doped biomaterials such as scaffolds doped with magnesium, silicon, or potassium, which stimulate angiogenesis and inflammation, and promotes osteogenic differentiation [24]. However, bioactive glass, 3D-printed scaffolds, and nanocomposite hydrogels are gaining popularity for their ability to deliver not just support but also biochemical signals through the controlled release of growth factors and gene vectors. Integration with stem cells and bioreactors is further advancing the potential of these biomaterials for functional bone tissue regeneration [25].

## 3. Physiochemical Parameters of the Intraosseous Environment

The intraosseous environment, crucial for sustaining bone health and function, is shaped by key physiochemical factors, including pH regulation, oxygen levels, ionic composition, calcium dynamics, phosphate balance, and trace mineral concentrations. These elements work in concert to regulate bone mineralization and support various cellular activities. Among these, the pH of the bone interstitial fluid (ISF) is particularly important, as it influences ion availability and the formation of hydroxyapatite, a primary mineral component of bone. Importantly, pH tightly controls phosphate levels, which directly affect hydroxyapatite precipitation and, in turn, the bone mineralization process [26].

The interstitial fluid (ISF) within bone contains a complex mixture of ions, including calcium, phosphate, carbonate, sodium, potassium, magnesium, and chloride, which interact to form various chemical species. Maintaining a delicate balance among these ions is essential for preserving the proper ionic strength of the ISF and preventing the unintended formation of mineral deposits. This balance ensures optimal conditions for bone health and function. Although detailed data on the oxygen tension in the intraosseous environment is limited, the oxygen levels are widely recognized as crucial for cellular metabolism and tissue function, including bone. Variations in oxygen tension can have a significant impact on bone cell activity and the mineralization process. Calcium ions, present in high concentrations in bodily fluids, are critical for maintaining bone density. However, their precise regulation is equally important in order to prevent undesired calcification in soft tissues. Proteins such as fetuin-A play a vital role in this regulation by stabilizing nascent mineral particles and inhibiting calcium phosphate sedimentation, thereby ensuring controlled mineralization and healthy bone formation [27]. Phosphate, in conjunction with calcium, is integral to the formation of hydroxyapatite, a fundamental component of bone mineralization. Its concentration is tightly regulated by pH, which plays a crucial role in influencing the mineralization process. This precise regulation ensures proper bone formation, maintenance, and overall skeletal health.

### Extracellular Matrix Microenvironment

The extracellular matrix (ECM) of bone is a complex and dynamic environment that plays an essential role in bone formation, regeneration, and the maintenance of overall tissue integrity. It is composed of both organic and inorganic components, which, together, provide structural support and regulate cellular functions. The organic portion of the bone ECM includes type I collagen, which imparts tensile strength, while the inorganic component, primarily hydroxyapatite crystals, provides the rigidity and hardness characteristic of bone [28]. Glycosaminoglycans are also present in the extracellular matrix (ECM) of bone, where they interact with various signaling pathways to influence bone remodeling. The ECM plays a central role in regulating the behavior of bone cells, including osteoblasts, osteoclasts, and mesenchymal stromal cells (MSCs). It affects key cellular processes such as adhesion, proliferation, differentiation, and responses to growth factors. These interactions are vital for maintaining bone homeostasis and facilitating remodeling, ensuring the continuous adaptation and repair of bone tissue [29]. ECM-based scaffolds are widely utilized in bone tissue engineering to facilitate bone regeneration. These scaffolds can be engineered to improve their osteoinductive, osteoconductive, and osteogenic properties, enhancing their effectiveness in repairing bone defects and supporting the regeneration of functional bone tissue [30].

The extracellular matrix (ECM) provides critical biochemical and biophysical cues that influence bone cell behavior. For example, the incorporation of hydroxyapatite within the ECM can enhance its angiogenic properties, which are essential for developing vascularized bone tissue models. This highlights the ECM’s role in supporting not only cellular activities but also the integration of vascularization in bone regeneration efforts [31]. In diseases such as cancer and hematologic malignancies, the extracellular matrix (ECM) plays a pivotal role in disease progression. By interacting with cancer cells, the ECM can influence their proliferation, survival, and behavior within the bone microenvironment, thereby shaping the dynamics of the disease and its impact on bone tissue [32]. ECM-modified biomaterial scaffolds and decellularized ECM scaffolds are being designed to replicate the natural bone environment. These advanced scaffolds aim to enhance integration with surrounding tissues and improve functionality, making them highly effective tools in promoting bone repair and regeneration [33]. A deeper understanding of the extracellular matrix (ECM) and its role in bone physiology and pathology can pave the way for more effective therapies for conditions such as osteoporosis and bone metastases. Additionally, this knowledge can improve the design and efficacy of bone grafts and regenerative treatments, offering significant advancements in the management and repair of bone-related conditions [34].

## 4. Regulatory Mechanisms

**Figure 4:**
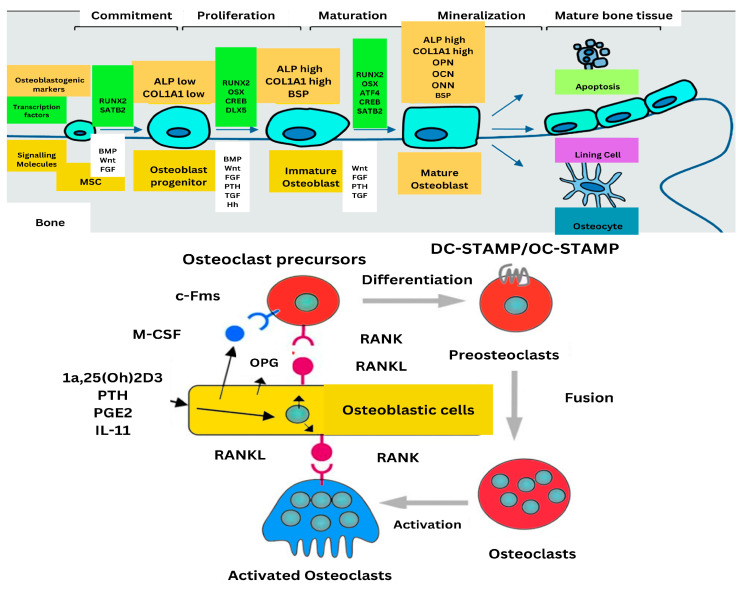
Regulation of bone formation and resorption: the upper panel illustration osteoblast differentiation and maturation, while the lower panel depicts osteoclastogenesis regulated by signaling molecules RANK/RANKL interaction and osteoblastic cell mediation.

Bone health and development are shaped by a complex interplay of regulatory mechanisms, hormones, cytokines, and signaling pathways. The parathyroid hormone (PTH) plays a pivotal role in maintaining calcium balance and facilitating bone remodeling. It promotes the secretion of monocyte chemoattractant protein-1 (MCP-1) by osteoblasts, which amplifies transforming growth factor-β (TGF-β) signaling, thereby contributing to its anabolic effects on bone. Although the specific roles of calcitonin and growth hormone were not detailed in the provided abstracts, these hormones are generally recognized for their influence on bone metabolism by regulating calcium levels and supporting bone growth. Additionally, sex hormones such as estrogen and testosterone are well-documented for their critical contributions to bone density and strength. Bone morphogenetic proteins (BMPs) and TGF-β are essential for bone formation and homeostasis. Figure 4 illustrated signaling pathways that regulate osteoblast differentiation and promote bone formation, with disruptions in BMP signaling being linked to disorders affecting bone mass [35].

Neuroendocrine influences, including sympathetic nervous system signals and stress hormones have profound effects on bone metabolism by regulating the activity of osteoclasts and osteoblasts. For example, increased catecholamines and glucocorticoids can stimulate bone resorption and inhibit bone formation, leading to diseases such as osteoporosis. Mechanical loading is instrumental in bone adaptation, with the osteocytes being able to perceive mechanical stress and communicate with the osteoblasts and osteoclasts via molecular signals such as the Wnt/β-catenin and RANK/RANKL/OPG signaling pathways. These pathways assist in governing bone formation and resorption relative to physical stress, facilitating efficient bone adaptation to different stresses. An investigation of these neuroendocrine and mechanical determinants would give a better insight into the regulatory processes controlling bone tissue homeostasis and adaptation.

### 4.1. Mechanical Stress and Mechanotransduction

Mechanical stress and mechanotransduction are key concepts for understanding how bones adapt to physical forces. Mechanical stress encompasses forces such as compression, tension, and shear, which act on bone tissue. Mechanotransduction refers to the process by which bone cells detect these mechanical forces and translate them into biochemical signals, triggering cellular responses that influence bone formation and remodeling. Bones are subjected to different types of mechanical stress, with fluid shear stress being particularly significant in influencing the behavior of bone cells [36]. Mechanical stress plays a vital role in preserving bone architecture and strength. It promotes bone formation while also regulating the delicate balance between bone formation and resorption [37]. Bone cells, especially osteocytes, possess mechanical stimuli. These receptors initiate signaling pathways, such as the Wnt signaling cascade, which play a crucial role in the bone’s adaptive response to mechanical loading [38].

Mechanosensitive channels, such as Piezo1 and Piezo2, are essential for bone development and the differentiation of osteoblasts. These channels mediate the response to mechanical forces by activating signaling pathways that involve key transcription factors, including NFAT, YAP1, and β-catenin [39]. Mechanotransduction triggers the release of signaling molecules that regulate the activity of osteoblasts and osteoclasts, playing a critical role in bone remodeling and adaptation [40]. Impairments in mechanotransduction can result in conditions such as osteoporosis, characterized by reduced bone mass and strength. These dysfunctions often stem from disrupted signaling pathways and increased osteocyte apoptosis. A deeper understanding of mechanotransduction pathways provides promising therapeutic targets for improving bone regeneration and addressing bone loss disorders [41].

### 4.2. Neuronal and Endocrine Interactions

**Figure 5:**
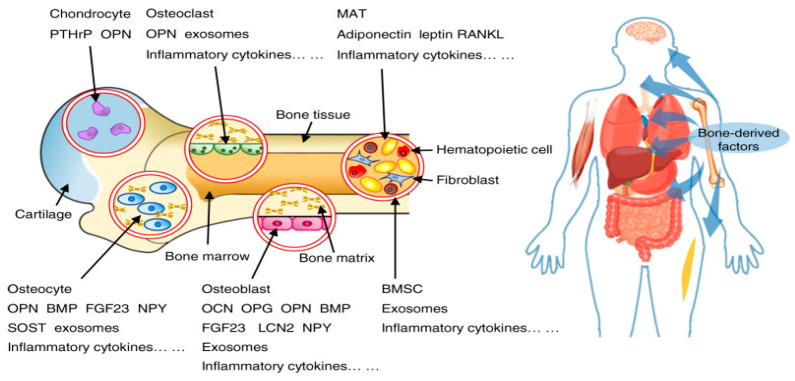
Illustration of neuronal and endocrine interactions with bone: highlighting the roles of the nervous system (via neurotransmitters and mechanical loading), the endocrine system (via hormones such as leptin, osteocalcin, and lipocalin-2), and their integration in regulating bone homeostasis and systemic metabolic balance [42].

Interactions between the nervous and endocrine systems with bones involve intricate regulatory mechanisms, which influence bone health and the overall metabolic balance. These interactions are vital for maintaining bone homeostasis, as indicated in Figure 5 [42]. The nervous system, through neurotransmitters such as serotonin and norepinephrine, plays a key role in regulating bone health. Specifically, the sympathetic nervous system can inhibit bone formation, while the parasympathetic system has the opposite effect, promoting bone formation [43]. Bones can detect mechanical loading and adapt its structure accordingly to minimize the risk of fractures. This adaptive process is significantly influenced by neuronal signals [44]. Bone secretes hormones like osteocalcin and lipocalin-2, which exert systemic effects. Osteocalcin plays a role in regulating insulin secretion and sensitivity, while lipocalin-2 influences appetite control by acting on the brain [45].

Hormones such as leptin, produced by adipocytes, influence bone metabolism through signaling via the hypothalamic relay, underscoring the endocrine system’s contribution to bone health. The neuroendocrine system, integrating both neuronal and hormonal pathways, plays a pivotal role in regulating bone homeostasis by modulating the processes of bone formation and resorption [43]. Pituitary disorders can impact bone remodeling and metabolism, demonstrating the significant role of pituitary hormones in maintaining bone health [46]. Thus, neuronal and endocrine interactions with bone are essential for sustaining bone health and maintaining systemic metabolic balance. The nervous system impacts bone through neurotransmitters, while bone functions as an endocrine organ by secreting hormones that regulate diverse physiological processes. Exploring these interactions offers promising avenues for developing new therapeutic strategies to address bone-related disorders.

## 5. Pathological Alterations in the Intraosseous Environment

**Figure 6:**
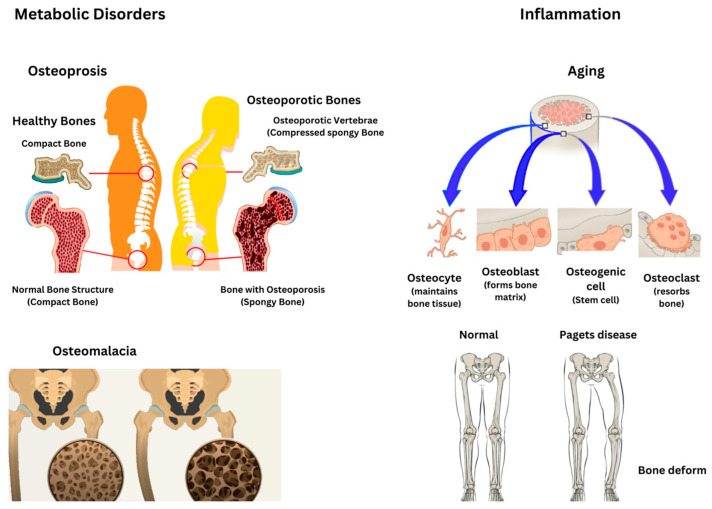
A visual overview illustrates the progression of pathological changes in bone health caused by metabolic disorders, inflammation, and aging. Healthy bones serve as the baseline, with pathways diverging to conditions such as osteoporosis, osteomalacia, Paget’s disease, chronic-inflammation-related bone damage, and age-associated bone loss. The summary highlights distinct mechanisms and outcomes, emphasizing how each condition disrupts bone structure and function.

Pathological changes in the intraosseous environment caused by metabolic bone diseases like osteoporosis, osteomalacia, and Paget’s disease result in specific alterations in bone mass, structure, and turnover as presented in Figure 6. Osteoporosis is marked by a reduced bone mass and compromised bone microarchitecture, which increases bone fragility and the risk of fractures [47]. Osteoporosis arises from an imbalance between bone resorption and formation, where osteoclastic activity exceeds osteoblastic activity, leading to a net loss of bone mass [48]. Similarly, osteomalacia stems from the defective mineralization of newly formed osteoid, resulting in softened bones and the development of characteristic loose zones. This condition can arise from nutritional deficiencies, genetic disorders such as X-linked hypophosphatemia, or other metabolic abnormalities [49]. Furthermore, Paget’s disease is characterized by excessive and disorganized bone remodeling, involving heightened osteoclast-mediated resorption followed by compensatory osteoblastic activity, ultimately leading to structurally compromised bone [50].

Paget’s disease has a significant genetic component, often associated with mutations in genes such as SQSTM1, and may also be influenced by environmental factors [51]. Paget’s disease can result in bone pain, deformities, arthritis, and fractures, with the rare potential for neoplastic transformations. A thorough understanding of these changes is essential for accurate diagnosis and effective management [52]. Inflammatory conditions, malignant transformations, and age-related changes have a profound impact on bone health. Inflammation disrupts bone remodeling by shifting the balance toward increased bone resorption. This process is driven by inflammatory cytokines and peptides, which modulate the expression of RANK and RANKL, key regulators of osteoclast and osteoblast activity [53]. Chronic inflammatory conditions such as rheumatoid arthritis and inflammatory bowel diseases are linked to bone loss or osteopenia. This is largely attributed to the prolonged activation of the immune system, which exacerbates inflammation and disrupts bone homeostasis [54]. Both acute and chronic inflammation influence bone repair, but dysregulated inflammation can result in heightened bone resorption and impaired bone formation [55].

Chronic inflammation in bone marrow can cause DNA damage in hematopoietic stem cells, potentially leading to bone marrow failure or leukemia. The inflammatory microenvironment plays a critical role in the development and progression of hematopoietic malignancies [56]. Inflammatory signals in the bone marrow can drive clonal selection, which, combined with aging and toxic insults, may contribute to malignant transformations. Aging is associated with chronic low-grade inflammation, termed inflammaging, which impairs osteoblastogenesis while promoting osteoclastogenesis, resulting in bone loss. The accumulation of senescent cells and their secretory phenotype (SASP) exacerbates chronic inflammation and bone aging, linking this process to both aging and cancer pathways. Aging also impacts the monocyte–macrophage–osteoclast and mesenchymal stem cell–osteoblast lineages, diminishing the osteogenic potential and complicating bone healing [57]. Table 2 summarizes all these disorders.

Inflammation, whether driven by chronic conditions or aging, significantly impacts bone health by enhancing bone resorption and inhibiting bone formation. This imbalance can result in conditions such as osteopenia and may also facilitate malignant transformations within the bone marrow. A deeper understanding of these processes is vital for devising strategies to prevent bone loss and promote bone health in aging populations.

Physiological bone remodeling is a tightly regulated ongoing process in which old bone is broken down by osteoclasts and rebuilt with new bone made by osteoblasts, with the aim of preserving bone mass and strength. The process is regulated tightly by mechanical loading, hormones, and numerous signaling pathways to prevent bone disease and ensure bone health and integrity throughout life. Bone resorption and formation are balanced in a healthy person, so bones can adapt to stress with the preservation of structural integrity, but the pathological remodeling of bones occurs when the delicate balance is disturbed by various diseases. Modern therapeutic approaches for metabolic and inflammatory bone diseases have advanced significantly, offering new strategies with which to manage conditions like osteoporosis, osteomalacia and Paget’s disease. For osteoporosis, treatments such as denosumab and romoszumab work by inhibiting osteoclast activity or stimulating osteoblast function, respectively, to increase bone mass and reduce fracture risk. However, the teriparatide recombinant form of parathyroid hormone promotes bone formation by osteoblasts, offering a novel approach to severe cases. In osteomalacia, where defective mineralization leads to softened bones, the mainstay of treatment is vitamin D and calcium supplements, often combined with calcimimetics and teriparatide to address mineralization defects and enhance bone strength. For Paget’s disease, bisphosphonates are routinely used to control excessive osteoclastic activity, but more novel therapies, including cathepsin K inhibitors, have been developed to directly target bone resorption at the molecular level. In chronic inflammatory diseases, including rheumatoid arthritis, biologic therapies such as TNF inhibitors and IL-6 inhibitors slow bone resorption by suppressing the immune system and treatments aimed at specific cytokines show promise for controlling inflammation-induced bone loss. These novel treatments, combined with a greater understanding of the role of genes and the practice of personalized medicine, assist in the management of these complicated bone diseases.

## 6. Emerging Technologies and Research Techniques

Emerging technologies and research techniques in bone studies are progressing rapidly, providing valuable insights into bone structure, function, and potential therapeutic approaches. Advanced methods such as high-resolution computed tomography (CT), synchrotron-based imaging, and ultra-high-field magnetic resonance imaging (MRI) offer a detailed visualization of bone’s 3D macrostructure and microstructure, enhancing the understanding of bone health and disease [58]. Techniques such as SPECT/CT, PET/CT, and whole-body MRI enhance the accuracy of diagnosing bone metastases and improve the monitoring of therapeutic responses [59]. Furthermore, techniques such as RT-PCR, FISH, and NGS play a vital role in diagnosing bone sarcomas and advancing personalized treatment strategies. These methods facilitate the identification of genetic alterations and support the development of targeted therapies, though further research is essential in order to address the challenges related to therapeutic resistance [60].

Three-dimensional culture models for bone microenvironment modeling provide a valuable tool for simulating the bone microenvironment, enabling the study of tumor interactions and the mechanisms of drug resistance. These models represent a promising alternative to animal studies in cancer research, with the potential to advance precision medicine approaches [61]. This technology has shown promising results in both human and animal studies for craniofacial bone repair. It enables the creation of 3D volumetric structures designed to support and enhance bone regeneration [62]. Advances in imaging technologies are enhancing the assessment of tissue-engineered bone grafts (TEBGs), which play a vital role in craniofacial bone repair. These innovations offer detailed insights into the dynamic healing process and the interactions between transplanted cells and host tissues [63]. Moreover, the integration of nanotechnologies into bone research is opening new opportunities for targeted diagnostics and therapeutics. Nanoparticles can be engineered to deliver drugs, growth factors, or gene-editing tools directly to the bone microenvironment, enhancing efficacy while minimizing systematic side effects. Nanosensors are also under development to detect early biochemical changes associated with bone diseases such as osteoporosis or metastasis, offering possibilities for real-time monitoring.

## 7. Conclusions

The intraosseous environment is essential to bone physiology, integrating mechanical, vascular, and cellular components that regulate bone health. Mechanosensitive ion channels such as TRP family members and Piezo ½ respond to mechanical loading and modulate bone remodeling through anabolic and catabolic pathways. Bone perfusion, sustained by intraosseous pressure, plays a key role in nutrient exchange, and it is implicated in disorders like osteoarthritis where vascular dysfunction contributes to disease progression. However, current IOP measurement techniques are limited to localized data, underscoring the need for advanced imaging and modelling tools that capture bone wide dynamics. Future research should focus on ion channel modulation as a therapeutic target the application of nanotechnologies and bioengineered scaffolds in bone regeneration, and the integration of omics and biomechanical data to explore systemic influences on bone health. However, these challenges, through multidisciplinary approaches, will be crucial for translating mechanistic insights into clinical strategies for diagnosing and treating bone-related diseases.

## Figures and Tables

**Table 1:** This Table explains different types of bone cells along with their origin and functions.

Cell Type	Function	Origin/Source	Key Signaling Pathways	Additional Notes
Osteoblasts	Synthesize the mineralized bone matrix; key role in bone formation.	- Chondrocytes within the growth plate. - Bone marrow stromal cells. - Quiescent bone lining cells. - Specific fibroblasts in craniofacial regions.	- Non-canonical Notch molecule Delta-like 1/preadipocyte factor 1 (Dlk1/Pref-1). - Wnt co-receptor Lrp5.	Short lifespan; requires renewal by preosteoblasts.
Osteoclasts	Specialized in bone resorption; degrades mineralized matrix and collagen by secreting acids and enzymes.	Derived from hematopoietic progenitors.	Regulated by signaling molecules from osteoblasts and osteocytes to maintain bone homeostasis.	The key for bone remodeling and calcium regulation.
Osteocytes	Central regulators of bone remodeling; coordinate osteoblast and osteoclast activity.	Differentiated osteoblasts are embedded in mineralized bone matrix.	Paracrine and endocrine signaling pathways.	Influence bone marrow fat, body composition, and energy metabolism; involved in bone diseases and therapeutic targets.
Bone marrow stromal cells (BMSCs)	Multipotent cells capable of differentiating into osteoblasts, chondrocytes, and adipocytes; are critical for bone regeneration.	Found in bone marrow stroma.	Regulated by various intracellular signaling pathways.	Linked to the development of osteosarcoma under specific genetic conditions.

**Table 2:** Key bone conditions: features, causes, and consequences.

Condition	Key Features	Causes	Consequences
Osteoporosis	Reduced bone mass and compromised bone microarchitecture, increasing bone fragility and fracture risk.	Imbalance between bone resorption and formation; osteoclastic activity exceeds osteoblastic activity.	Increased fracture risk due to fragile bone microarchitecture.
Osteomalacia	Defective mineralization of osteoid leads to softened bones and characteristic loose zones.	Nutritional deficiencies, genetic disorders like X-linked hypophosphatemia, or other metabolic abnormalities.	Softened bones and the development of loose zones.
Paget’s Disease	Excessive and disorganized bone remodeling with elevated osteoclast and osteoblast activity, leading to structural compromise.	Genetic mutations (e.g., SQSTM1) and environmental factors.	Bone pain, deformities, arthritis, fractures, and rare neoplastic transformations.
Aging	Chronic low-grade inflammation (inflammation) impairs osteoblastogenesis and promotes osteoclastogenesis, causing bone loss.	Senescent cells and their secretory phenotype (SASP), diminished osteogenic potential, and inflammation.	Bone loss, impaired bone healing, and links to cancer pathways.

## Data Availability

No new data were created or analyzed in this study.
